# Band transport across a chain of dopant sites in silicon over micron distances and high temperatures

**DOI:** 10.1038/srep19704

**Published:** 2016-01-21

**Authors:** Enrico Prati, Kuninori Kumagai, Masahiro Hori, Takahiro Shinada

**Affiliations:** 1 Istituto di Fotonica e Nanotecnologie, Consiglio Nazionale delle Ricerche, Piazza Leonardo da Vinci 32, I-20133 Milano, Italy; 2 Graduate School of Science and Engineering, Waseda University, 3-4-1 Ohkubo, Shinjuku, Tokyo 169-8555, Japan; 3 Graduate School of Science and Engineering, University of Toyama, 3190 Gofuku, Toyama 930-8555, Japan; 4 Center for Innovative Integrated Electronics System, Tohoku University 468-1 Aramaki Aza Aoba, Aoba-ku, Sendai, Miyagi, 980-0845, Japan

## Abstract

Macroscopic manifestations of quantum mechanics are among the most spectacular effects of physics. In most of them, novel collective properties emerge from the quantum mechanical behaviour of their microscopic constituents. Others, like superconductivity, extend a property typical of the atomic scale to macroscopic length scale. Similarly, features of quantum transport in Hubbard systems which are only observed at nanometric distances in natural and artificial atoms embedded in quantum devices, could be in principle extended to macroscopic distances in microelectronic devices. By employing an atomic chain consists of an array of 20 atoms implanted along the channel of a silicon transistor with length of 1 *μ*m, we extend to such unprecedented distance both the single electron quantum transport via sequential tunneling, and to room temperature the features of the Hubbard bands. Their observation provides a new example of scaling of quantum mechanical properties, previously observed only at the nanoscale, up to lengths typical of microelectronics, by opening new perspectives towards passage of quantum states and band engineering in silicon devices.

Differently from other macroscopic manifestations of quantum mechanics^1^,^2^,^3^,^4^,^5^,^6^ for which novel collective properties emerge^7^, long range quantum transport, like superconductivity^8^, could extend properties typical of the atomic scale to microelecronic length scale. The creation of quantum channels^9^ obtained by arrays of atoms^10^,^11^ acting as one dimensional atomic chains in silicon devices for quantum state transmission may unleash new possibilities for information processing^12^,^13^,^14^, ranging from molecular scale circuits^15^,^16^,^17^ to quantum processing^18^, quantum cellular automata and computing^19^. In the last few years atomic scale solid state devices have been adopted as a promising experimental platform for quantum state transmission^20^. While quantum transport, Hubbard band formation and coherent transport from adiabatic passage of electrons have been observed at nanometric length scale by involving few quantum states^20^, they could in principle be extended to longer distances in devices equipped with a sufficiently ordered one dimensional array. We reported on quantum transport and Hubbard band formation in arrays of few As and P donors implanted in silicon nanometric sized devices^10^. However, for exploiting atomic chains for quantum state transport across quantum circuits, the same properties have to be extended to inter-device distances. We report on silicon transistors of length up to 1 *μ*m where the drain and the source regions are connected by a chain of phosphorous atoms creating a conductive channel in the silicon bandgap. The resulting atomic chain shows the quantum properties of conductance peaks and Hubbard band formation^21^ previously reported at nanometric distances and at cryogenic temperature^10^, at macroscopic distances and room temperature respectively. Here we report on the first observation of conductance peaks of an atomic chain of 1 *μ*m length and Hubbard band formation from cryogenic to room temperature. Precisely, we show that the ground states of the donors in the proximity of the silicon interface contribute to form a set of localized states supporting multiple sequential tunneling, while their excited states contribute to form the upper Hubbard band, which exhibits different activation energies according to the temperature regime.

For the sake of consistency, all the measurement reported in the main text refer to the same sample a1. Additional results from other samples are reported in the Supplementary Information.

Artificial atoms^9^, as well as donor atoms in silicon^17^,^22^,^23^,^24^,^25^,^26^,^27^, are conveniently treated as Hubbard systems^21^ in which onsite Coulomb repulsion is able to create a gap^28^. Even if a number of properties of solids are accounted for in terms of Bloch bands, while Coulomb interactions between electrons are neglected, band theory fails for materials with low magnetic-ordering temperatures but large insulating gaps^29^. Mott^28^ suggested that interaction-induced insulators are better described in real space, in which the properties emerge from localized electrons bound to atoms with partially filled shells. Two bands are originated and split by the energy cost to add a second charge on the same atomic site. Such systems are described by Hubbard bands in the strongly correlated (or atomic) limit. Electronic density of states and electron occupancy associated to donors in silicon is accounted for by Anderson-Mott physics. Single-band Hubbard Hamiltonian expresses the competition between two energy scales, consisting of the kinetic energy, which depends on the overlap between electronic wave functions on neighboring lattice sites, and the Coulomb energy *U*, which returns the strength of the onsite Coulomb repulsion between two electrons. The Mott physics is captured by the Hubbard Hamiltonian:


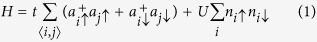


where 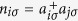, *t* is the hopping matrix element and *U* is the on-site repulsion energy (*U* > 0).

Large arrays of quantum dots connected in a series by tunnel coupling have been treated in the past as a one-dimensional lattice and quantum transport under dc bias has been predicted^30^. In the case of fermions in a linear array of *N* elements, generated for instance by a series of quantum dots or donor atoms (Fig. 1), in which only first neighboring sites interact, the whole system is treated as a *single quantum system* (Fig. 2a) and many-body eigenstates have been calculated by exact diagonalization (exemplified in Fig. 2b) leading to single electron tunneling effect^9^,^31^. In the case of small interdot coupling with respect to the intradot Coulomb repulsion, a condition which holds for P donors in Si, the density of states consists in two sets of *N* peaks in the limit of *T* → 0 K, which merge in two bands by raising the temperature (red dashed line), reminiscent of the mentioned Hubbard bands (Fig. 2b), or by increasing the overlap integral between neighboring sites. The addition energy separating the N peaks, not to be confused with standard Coulomb blockade peaks typical of a single quantum dot, generated by the eigenstates formed by the ground states *D*_0_ partially hybridized at the silicon bottom interface, is expected of the order of few meV^30^,^31^. These facts were experimentally confirmed for a small number of atoms^10^ for which the transition from peaks of conductance at 4.2 K to the first Hubbard band was observed by raising the temperature, while the upper Hubbard band was already formed at 4.2 K and above.

Transistors used in the experiments are fabricated on (100) silicon-on-insulator (SOI) substrate with 125-nm-thick buried oxide (BOX), acting as a back-gate oxide (Fig. 1b). The nominal channel length is 1 *μ*m, the width and thickness of the channel are 200 nm and 90 nm, respectively (see Supplementary Information). Figure 1c shows an idealized graphical representation of the potential in the channel of the device after atom implantation.

As the condition to observe collective states across the system is granted by non-zero coupling between neighboring sites, fluctuations around the ideal position of the implanted dopants do not represent a limitation within some extent. Formation of extended states is not prevented by deviations from the ideal condition of identical energies and tunnel couplings^32^. By considering that the fluctuations granted by single-ion implantation (SII) method^33^ are smaller than the distance between neighboring sites and that two atoms are implanted in each site, the partially disordered chain is expected to grant electron transport, as previously observed in smaller SII devices^10^. Silicon wires of length of 280 nm with array of 7 active randomly diffused dopants have been reported to exhibit both lower and upper Hubbard bands and conductance oscillations in the lower band^34^.

The *N* = 20 atomic chain with length of 1 *μm* behaves as a single quantum system at 4.2 K, as observed from the conductance peaks in the subthreshold region of the transistor. As expected from the theory, sub-threshold electron transport at 4.2 K reflects two bands partially overlapped (Fig. 2b) corresponding to the case marked with the green hexagon of Fig. 2a. The first manifests as a set of conductance peaks determined by the collective states formed by the ground states *D*^0^ of the donors partially hybridized with the interface states^35^, corresponding to sequential electron tunneling. The second band, because of the larger extension of electron wavefunction of *D*^−^ states (forming the upper Hubbard band already above a density equivalent to 3 × 10^15^ cm^−3^ in bulk^36^) is a full thermally activated band, based on variable range hopping between localized sites, as already reported in ref. 10 for samples of smaller length. As described later, thermal activation in the lower Hubbard band is observed at higher temperature. Differently, the variable range hopping involving the more extended *D*^−^ states overcomes the sequential electron tunneling at the base temperature of the experiment. At low gate voltages, the *I* − *V*_*g*_ characteristics shows a series of conductance peaks up to −1 V (Fig. 2c), while at high gate voltages it shows a flat band corresponding to the upper Hubbard band, which partially overlaps with the lower band (the series of peaks at 4.2 K), as shown in Fig. 2b. The conductance peaks generated by the collective states formed by the donors, whose number corresponds to the number of donors (SI Fig. 2a–c), are shown in Fig. 2c while the absolute value of the current stability diagram is displayed in Fig. 2d, by revealing a peak separation of the order of few meV. The correspondence between the number of conductance peaks and the number of implanted donors has been confirmed in shorter samples (SI Fig. 2d,2e). The combination of a larger extension of the *D*^−^ state with the possibility to activate variable range hopping, differently from lower Hubbard band, allow to observe the upper Hubbard band^28^ at 4.2 K. The maximum conductance of the sequential tunneling observed by the peaks is, once the upper Hubbard band is subtracted (SI Fig. 2c), of the order of *σ* ≈ 10^−4^  ÷ 10^−2^ *μ*S, which depends on both the sample-specific coupling of the wavefunction of the bound electrons with the drain and the source electron wavefunctions, like previously reported values in other randomly diffused or deterministically implanted samples^10^,^11^,^22^. The bias voltage of few meV employed for the measurements allows the observation of isolated conductance peaks associated to sequential tunneling processes. By raising *V*_*ds*_ the heating of the electron system broadens the upper Hubbard band left tail so both sequential tunneling processes and hopping processes occur in parallel. The formation of a single quantum state across the device caused by the chain of P atoms makes the dephasing length *L*_*ϕ*_ sufficiently long to make possible the observation of quantum transport even if the length of the channel *L* exceeds the lengths typical of interdevice distances of microelecronics. In addition, it is worth to mention that in those voltage and temperature ranges where diffusion is observed, the system consists of a 1-dimensional disordered chain of impurities close to the edge of the metal insulator transition (MIT)^37^. By approaching the critical point the correlation diverges and the electron system wavefunction becomes multifractal, so correlation spreads ideally at all length scales^38^.

Now, we turn to the thermal activation of the electron transport explored by the temperature dependence up to room temperature. The analysis of thermal activation demonstrates both that the system evolves consistently with Anderson-Mott physics of Si:P as the temperature is increased from base temperature, and that the upper Hubbard band is maintained when the temperature is raised up to room temperature. The latter constitutes a novel macroscopic effect made possible by the 1-dimensionality of doping of the channel, never previously reported in randomly doped transistors. In Fig. 3a, the thermal effects on the electron transport through the atomic chain is reported. The transconductance characteristics *σ* = *I*_*DS*_/*V*_*DS*_ of the transistor versus the gate voltage *V*_*G*_ is measured at several temperatures up to 274 K by applying *V*_*ds*_ = 2.505 mV. Let’s first consider the activation of the lower Hubbard band from the conductance peaks regime. By raising the temperature, the single electron quantum transport through the collective states formed by the *D*^0^ states observed at 4.2 K is progressively dominated by the thermally activated processes between localized states so the lower Hubbard band appears. According to the convention introduced by Mott^28^, the activation energy of this process is called 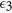. As discussed, the distance from the Si/SiO_2_ interfaces of the donors implanted through SII varies randomly within a limited range in the proximity of the bottom interface. Consequently the potential landscape created by the atoms of the atomic chain, assisted by the enlargement of the electron wavefunction of *D*^0^ states by the hybridization with interface states in silicon^35^, creates a band ascribed to the interdot coupling between neighboring sites, capable to support extended states from neighboring sites and single electron transport through a set of about 20 states. On the contrary, at sufficiently high temperature, the atomic chain also acts as a random potential energy in the sense of Anderson^39^. In order to extract the activation energy, the resistivity *ρ* is plotted in logarithmic scale, being 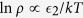 and 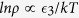 for the upper and the lower Hubbard bands respectively^28^, where *k* is the Boltzmann constant. The linear fit of the slope of the lnρ as a function of 1/*T* returns the activation energies 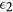 and 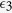 after dividing by *k*. The lower Hubbard band emerges (Fig. 1c, region marked with the blue circle) by raising the temperature, by returning an activation energy of 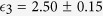 (Fig. 3c), a small value consistent with previous reports^10^,^28^. Such activation energy is some mean of the energy interval between nearest-neighbor sites. The effect on the flat band attributed to the upper Hubbard band, typical of all the samples doped with the same implantation pitch (see Supplementary Information Figure S1c on a nominally identical sample and Supplementary Information Figure S2d on a sample with half length and half implanted donors), is even more remarkable as it remains observable from 4.2 K to 274 K. The reason of the remarkable difference from randomly diffused devices, for which doping just produces a shift of the threshold voltage towards lower voltages, and not a sub-threshold flat band, is ascribed to the dimensionality of the density of states (DoS). Differently from the latter, for which the rapidly growing 3-dimensional DoS as a function of the energy - scanned by the gate voltage - allows a large number of excitations within the impurity band, observed as a lower threshold voltage, in the 1-dimensional case the DoS realized by the SII atomic system is flat (see Fig. 2a, below). Consequently, only few states are available within the band and the amount of excited electrons is highly constrained. The effect may be further assisted by incomplete ionization mechanism, which appears at room temperature in such average doping density range for Si:P^40^. This is also confirmed by observing the evolution of the upper Hubbard band at room temperature when the implantation pitch is changed, by returning a 3-dimensional metallic character in samples whose density is higher (see Supplementary Information Figure S1b, 25 nm pitch).

Above 250–260 K, the transport in the silicon conduction band overcomes the Hubbard band transport at high gate voltage. This is determined by both the progressive decrease of the threshold voltage as the temperature is raised (which can be extracted to be around *V*_*T*_ ≅ 2 V at room temperature, see inset of Fig. 2b), and the thermal activation of transport in the conduction band.

Differently from Hubbard bands, for which transport occurs along the atomic chain, transport in the conduction band of the silicon channel occurs at the Si/SiO_2_ interface at the side of the back gate, when sufficiently positive voltage is applied. As the lower and upper Hubbard bands partially overlap, two activation energies are expected to be associated to the upper band according to the temperature range^28^. The activation energy 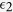 refers to *nearest-neighbor hopping* by electrons at the Fermi energy while 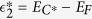 where 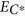 is the energy separating the localized from the extended states in the upper Hubbard band, and it consists of the activation energy for *non-hopping conduction*. In addition, according to Mott theory^28^, the upper Hubbard band is predicted to exhibit a third behaviour at very low temperature, consisting in *variable range hopping* below a critical temperature *T*_*kink*_ which is linear as a function of 1/*T*^*α*^ where *α* = 1/(*d* + 1) and *d* is the dimensionality of the system. The transition from variable range hopping at the base temperature to thermally activated nearest-neighbour hopping between localized states at the Fermi energy is shown in (Fig. 3d). The corresponding activation energy of the upper Hubbard band at low temperature is 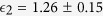 meV, a small value consistent with previously reported values^10^,^28^. Variable range hopping below a critical *T*_*kink*_ was observed in previous experiments with few donors^10^. Similarly, we observe the kink at around *T*_*kink*_ = 8.6 ± 0.5 K, below which the variable range hopping sets in. It is remarkable that below *T*_*kink*_, the logarithm of resistance exhibit a linear trend as a function of *T*^*α*^ with *α* = 1/2 (inset of Fig. 3d), which returns dimensionality *d* = 1, consistently with the quasi 1-dimensional atomic chain. In general, Anderson localization in bulk semiconductors does not require attractive potentials, but rather comes about by trapping of the particle in cages at sufficiently high density of scatterers. In the soft potential landscape created by the array of atoms, the delocalization can be achieved not only by decreasing the density of scatterers, but also by increasing the energy of the scattered particles^41^. Therefore, at high temperature the localization of the upper Hubbard band is expected to be lost and the mentioned higher activation energy typical of excitations from the localized to delocalized states at the mobility edge is expected. By raising the temperature from about 80 K to room temperature (Fig. 3b), this prediction is beautifully confirmed, as, being 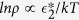, the measured activation energy of the upper Hubbard band in this temperature range is 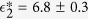 meV (the * suffix is used to denote high temperature regime), close to experimental value of 7.6 meV determined in ref. 36. In addition to transport measurements, we report on noise spectrum measured in correspondence of the Hubbard band at room temperature, where diffusion along the weakly disordered chain takes place. In the past, the noise spectrum of 1-d electron diffusion has been predicted to show an asymptotically 1/*f*^1/2^ trend^42^,^43^,^44^,^45^,^46^,^47^, which significantly differs from 1/*f* noise typical of low frequency noise spectrum of a transistor. In Fig. 4, we show that at room temperature, above the threshold voltage, the conduction band related transport exhibits standard 1/*f* noise together with 1/*f*^1/2^ noise dominating at higher frequency, which remains the only noise spectrum by setting the Fermi energy within the Hubbard band. Such noise spectrum has never been reported in a transistor, it is caused by the impurity band, and it cannot be explained with standard theory of transistors. Even if not conclusive *per se*, such effect may provide an independent manifestation of the 1-dimensionality of the atomic chain in addition to quantum transport and variable range hopping measurements.

To conclude, transport characterization at cryogenic temperatures of an atomic chain of ~20 phosphorous atoms implanted as an array along the channel of a silicon transistor with length of 1 *μ* shows the formation of extended states supported by *D*^0^ ground states of the atoms close to interface, while the *D*^−^ states contribute to form the upper Hubbard band. The extended states are observed as sequential single electron tunneling across the array of atoms at 4.2 K, which covers the unprecedented distance of 1 *μ*m. Such length corresponds to interdevices distances, preparing the ground for long distance quantum state transport in solid state quantum information processing. At such temperature, 1-dimensional variable range hopping is thermally activated in correspondence of the upper Hubbard band. By raising the temperature above 4.2 K, both the thermally activated lower and the upper Hubbard bands are observed, with activation energies 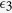 and 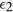 respectively, of few meV. By raising the temperature above ~80 K, the thermal assisted hopping at the Fermi energy is replaced by non-hopping excitations to mobility edge so electrons of the upper Hubbard band delocalize and exhibit a higher activation energy 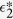 up to room temperature. The 1-dimensionality is independently suggested by the observation of 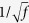 noise associated to the Hubbard bands. Our results extend few atoms physics to arrays of tens of atoms at interdevice distances, for which quantum state transport is pursued, together with the observation of 1-dimensional Hubbard bands up to room temperature. Such findings scale up to macroscopic distances the quantum mechanical mechanism usually involving few atoms, and open new approaches for the investigation of quantum state transport and band engineering in strongly correlated condensed-matter systems.

## Additional Information

**How to cite this article**: Prati, E. *et al.* Band transport across a chain of dopant sites in silicon over micron distances and high temperatures. *Sci. Rep.* **6**, 19704; doi: 10.1038/srep19704 (2016).

## Supplementary Material

Supplementary Information

## Figures and Tables

**Figure 1:**
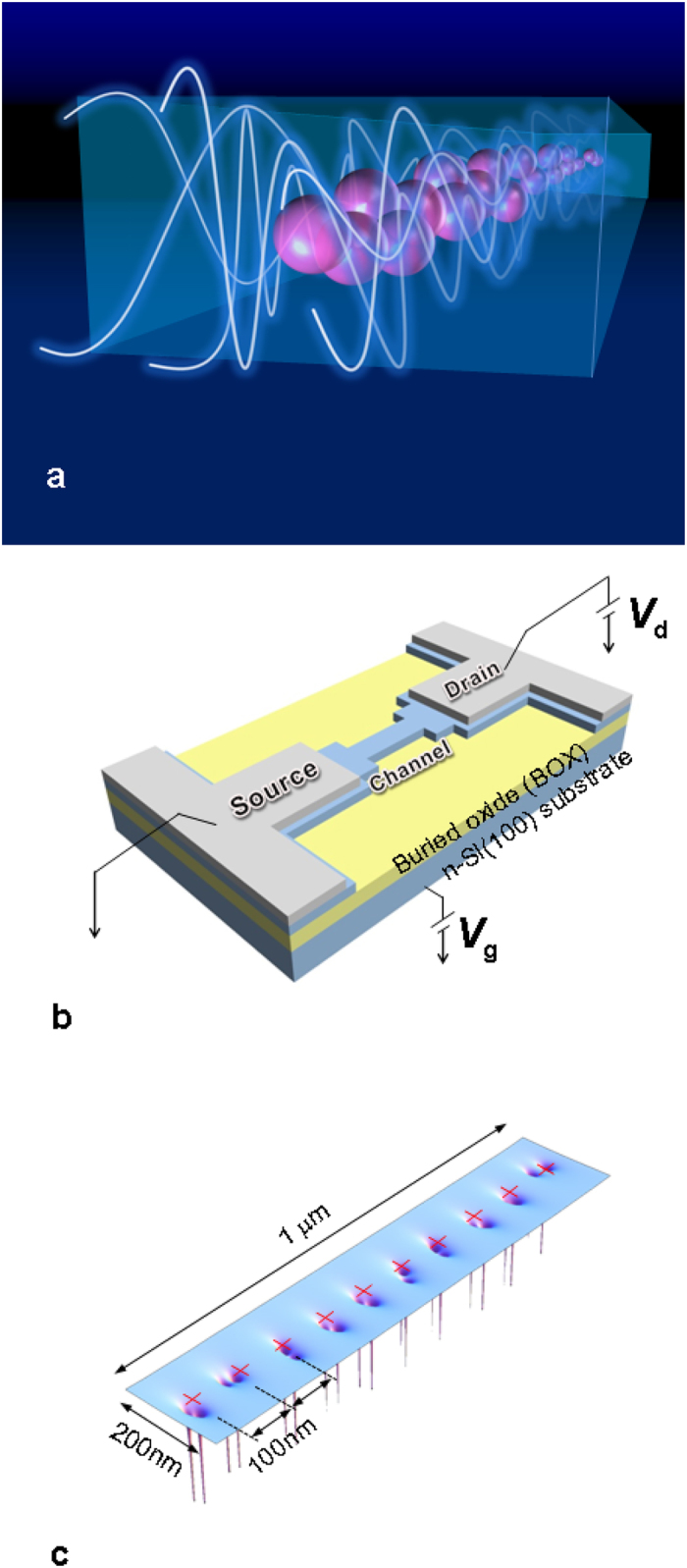
Single ion implanted devices. The experimental devices and calculated potential distributions in the channel regions. (**a**) An ideal representation of the electron tunneling across the 1 *μm* device thanks to the collective electronic states created by the donor array. (**b**) Geometry of the device structure for controlling transport by controlling the dopant position via single-ion implantation. (**c**) An idealized representation of the potential distributions in the 20 phosphorous donors distributed along the channel of the sample. The red crosses indicate the target positions.

**Figure 2:**
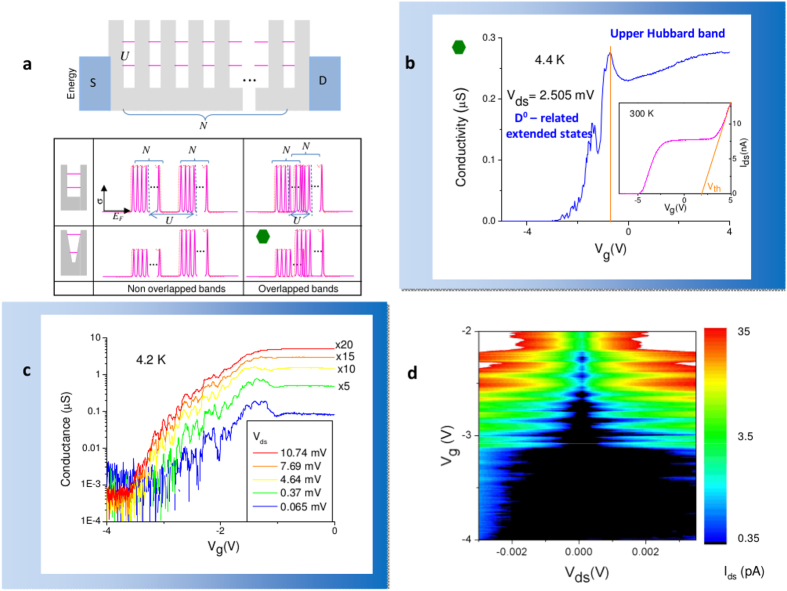
Quantum transport across an array of 20 implanted atoms. (**a**) energy diagram of an ideal array of *N* natural or artificial quantum dots, with identical charging energy *U*. The system is predicted to support extended states providing, in the limit *U*  *t*, two sets of N conductance peaks, with center-to-center separation *U* (pink). The conductance characteristics starts to reflect the formation of Hubbard bands as either the chain gets longer^31^, or the temperature is raised. Four idealized possible conditions may take place, providing different organization of conductance peaks. The ratio between the bandwidth *B* and *U* determines the overlap between the two sets of peaks (bands), while the shape of the potential determines a larger conductivity for more overlapped two electron states, like *D*^−^ 36). (**b**) Transconductance *σ* of the device probed at 4.4 K measured at *V*_*ds*_ = 2.505 mV. *V*_*G*_ scans the Fermi energy of the system according to the sketch (**a**). At low *V*_*G*_, on the left side of the orange line around −1 V, about 20 conductance peaks are observed. On the right side, because of the higher overlap of the *D*^−^ states^36^, the flat upper Hubbard band is visible. The orange line ideally terminates the voltage range where conductance peaks via *D*_0_-related states occur. As the two bands are partially overlapped, the conductance peaks are raised at the right side by the left tail of the upper band (for the discrimination of the two, and for a device of half length and atoms, see Supplementary Information Figure S2a,b,c and d). Inlet: extraction of the threshold voltage at room temperature, at *V*_*ds*_ = 2.505 mV. (**c**) the conductance peaks measured at 4.2 K at different biasing voltages. The effects of the heat dissipation when the current *I*_*ds*_ increases by raising the drain voltage *V*_*ds*_ is observed as a broadening of the peaks, as well as a higher thermal activation of the upper Hubbard band above −1 V on the right. (**d**) stability diagram of the conductance peaks, revealing the occupation by the first electrons. An addition energy of few meV is observed between consecutive electron filling of *D*^0^-related states, as expected.

**Figure 3:**
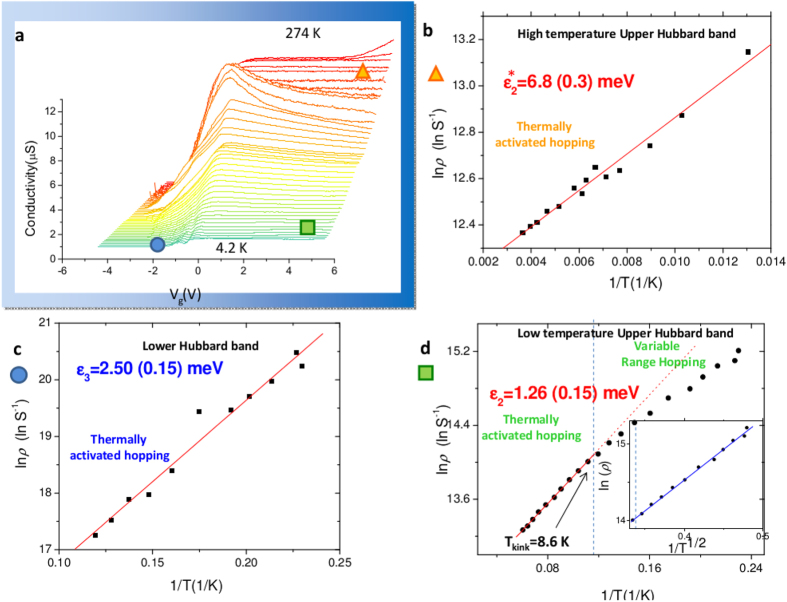
Thermal activation of transport. (**a**) the transconductance as a function of the gate voltage *V*_*g*_, from 4.2 K to 274 K (room temperature). The conductance peaks at low gate voltage are rapidly dominated by the thermally activated processes, so the lower Hubbard band builds up (blue dot). At high gate voltage, the low temperature region shows the upper Hubbard band at low temperature (green square) which remains visible up to room temperature (orange triangle). Only at the highest temperature the above-threshold linear regime of the device involving the *Si*/*SiO*_2_ interface conduction band appears. The bias voltage *V*_*ds*_ = 2.505 maximizes the signal-to-noise ratio by granting the approximation of the electron temperature with the nominal temperature. The activation energies have been extracted at voltages out of the overlap range in order to refer to consistent data. (**b**) Above about 70–80 K (corresponding to *kT* = 5.8–6.6), the thermal activation of the upper Hubbard band returns an activation energy 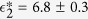 meV at 4 V (the error is calculated by standard linear regression), corresponding to activation from localized to delocalized states within the upper Hubbard band. (**c**) The thermal activation of the lower Hubbard band returns 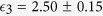 meV at *V*_*g*_ = −2 V. The high accuracy temperature control is made possible by the smooth gradient of He vapors in the dewar combined with the vertical control of the height of the device. (**d**) At low temperature, according to Anderson theory^39^ and similarly to shorter arrays^10^ the upper Hubbard band as an activation energy of 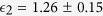 meV above the kink (here at 8.6 K), while at lower temperatures variable-range hopping is observed. The linearity of *lnσ* ∝ −*T*^−1/2^ (inset, blue line) is consistent with 1-dimensional variable-range hopping. The fitting function at the two sides of the kink was obtained by comparing a single linear, a single square root, a single fourth root, and their combinations at the left and the right of the kink.

**Figure 4:**
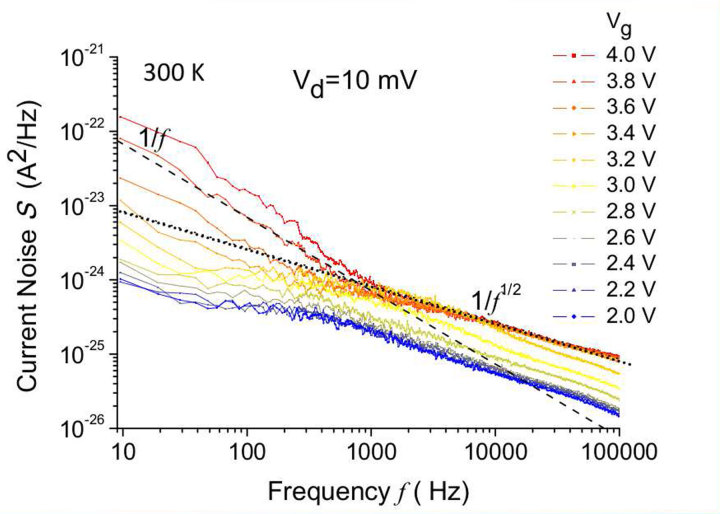
Noise spectrum. The noise spectrum is measured at *V*_*ds*_ = 10 mV at room temperature by using a correlation amplifier^48^ which eliminates the noise of the drain and the source contacts, capable to provide the electronic noise of the channel of the transistor. When the conduction band is populated (around *V*_*g*_ = 4 V) the low frequency noise is 1/*f* which is typical of transistors, but unprecedented 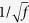 noise dominates at higher frequency, thanks to the parallel channel opened by the Hubbard band. When the gate voltage is lowered to 2 V and the transport in the conduction band is progressively reduced and turned off, the 1/*f* noise disappears. The transport occurs in the Hubbard band and the only 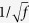 noise is observed, consistently with 1-dimensionality of the atomic array^47^,^49^.
