# Supplementary material for: Band transport across a chain of dopant sites in silicon over micron distances and high temperatures
Source: Sci Rep. 2016 Jan 21;6:19704. doi: 10.1038/srep19704 (PMC4726244; doi:10.1038/srep19704)
Supplement: Supplementary Information [file srep19704-s1.pdf]

Supplementary Information: Band transport across a chain of dopant sites in silicon over micron distances and high temperatures

Enrico Prati,* Kuninori Kumagai, Masahiro Hori, and Takahiro Shinada†

*Electronic address: enrico.prati@cnr.it

†Electronic address: shinada@cies.tohoku.ac.jp

I. THE SINGLE-ION IMPLANTATION METHOD

In the SII method [1–3], single ions are extracted by chopping a focused ion beam (FIB) using a small aperture and high-frequency beam deflection. The number of implanted ions is individually controlled by detecting secondary electrons emitted from a target or current change in a transistor upon the incidence of a single ion [4]. Because the number of ions extracted by chopping obeys a Poisson distribution, the number of ions extracted by a single chop must be sufficiently less than one. We have evaluated the number of extracted ions to be 0.001 ions/chop, given a beam current of 0.5 pA, a beam chopping transient time of 25 ns and an objective aperture of 20 μm. Thus, 1000 chops will extract a single ion on average; namely, the number of extracted ions per single chop (0.001 ions/chop) is sufficiently small so that multiple ions are essentially never extracted. A broad range of ion species, such as Be, B, Si, P, Ni, Cu, Ga, Ge, As, Pd, Pt and Au, can be individually implanted with an aiming precision of 50 nm and a probability of 50% (or potentially higher). The achieved secondary electron detection efficiency is 90% due to the low probability of secondary electron emission. This efficiency has been enhanced to almost 100% by increasing the number of secondary electrons and controlling the substrate bias voltage (V_g) [5]. The secondary electron detection efficiency increased as V_g became increasingly negative and was found to be as high as 20% at -5 V. This enhancement enables the reliable detection of single dopants.

II. SINGLE ION IMPLANTED TRANSISTORS

The implantation pitch falls in the density regime of $10^{16} - 2 \times 10^{17}$ cm^{-3} of bulk silicon, at which donor clusters in bulk are formed and Hubbard bands start to form in bulk from D^0 state of the donors [6], while upper Hubbard band behaviour is already present from 3×10^{15} cm^{-3}[7]. By considering intrinsic silicon of the channel to have residual donor density of the order of 10^{15} cm^{-3}, about 0-2 additional not-intentionally implanted atoms may participate to the array. As the distribution of the implantation depth is centered around 20-25 nm, we estimate that 0-2 donors may escape beyond the bottom interface, so we assume that the total number of donors is $N = 20 \pm 2$. As the distribution is centered at about 20-25 nm from the bottom Si/SiO_2 interface, some hybridization of the electron wavefunction with interface states [8] is expected to enlarge the effective radius and to assist overlap between the tails

of the wavefunction of neighboring sites to increase also for D_0 states. The channel is in contact with highly doped n-type source and drain regions and it is controlled by a gate bias (V_g) from the substrate through the buried oxide. The device exhibits accumulation-mode n-type transistor operation. The phosphorous pairs of ions were implanted at 60 keV into the channel with 90 nm thick through the surface oxide with 10 nm thickness. The average depth of the implanted phosphorous ions is calculated to be 65-70 nm in the silicon layer. Thus the donors are expected to distribute around the depth of 20-25 nm from the interface on the back side. The not-implanted samples and insufficient density implanted samples do not exhibit either conductance peaks or any kind of transport below the threshold voltage (see Figure S1a, S1b, S1d, S1e). Two dopants were randomly placed at each site in the active channel region through single-ion implantation with an aiming accuracy of 50 nm with a probability of 50%.[9] To electrically activate the implanted ions, the samples were lamp-annealed at 900 °C for 1 min in N_2. According to Fick's law of diffusion, the additional consequent diffusion length is of about 10 nm.

---

[1] Matsukawa, T., Shinada, T., Fukai, T. & Ohdomari, I. Key technologies of a focused ion beam system for single ion implantation. *J. Vac. Sci. and Technol.* **B 16,** 2479-2483 (1998).

[2] Shinada, T., Okamoto, S., Kobayashi, T. & Ohdomari, I. Enhancing semiconductor device performance using ordered dopant arrays. *Nature* **437,** 1128-1131 (2005).

[3] Jamieson, D. N. et al. Controlled shallow single-ion implantation in silicon using an active substrate for sub-20-keV ions. *Appl. Phys. Lett.* **86,** 202101 (2005).

[4] Shinada, T. et al. A reliable method for the counting and control of single ions for single-dopant controlled devices. *Nanotechnology* **19,** 345202 (2008).

[5] Hori, M. et al. Enhancing single-ion detection efficiency by applying substrate bias voltage for deterministic single-ion doping. *Appl. Phys. Expr.* **4,** 046501 (2011).

[6] Thomas, G. A., Capizzi, M., DeRosa, F., Bhatt, R. N. & Rice, M. T. Optical study of interacting donors in semiconductors. *Phys. Rev.* **B 23,** 5471-5494 (1981).

[7] Norton, P. Formation of the upper Hubbard band from negative-donor-ion states in silicon.

Phys. Rev. Lett. **37,** 164-168 (1976).

[8] Lansbergen, G. P. et al. Gate-induced quantum-confinement transition of a single dopant atom in a silicon FinFET. Nature Phys. **4**, 656-661 (2008).

[9] Shinada, T., Koyama, H., Hinoshita, C., Imamura, K., Ohdomari, I., Improvement of Focused Ion-Beam Optics in Single-Ion Implantation for Higher Aiming Precision of One-by-One Doping of Impurity Atoms into Nano-Scale Semiconductor Devices, *Jpn. J. Appl. Phys.* **41**, L287-290 (2002).

[10] Mott, N. F. & Twose, W. D. The theory of impurity conduction. *Advances in Physics* **10**, 107-163 (1961).

[11] Prati, E., Hori, M., Guagliardo, F., Ferrari, G. & Shinada, T. Anderson-Mott transition in arrays of a few dopant atoms in a silicon transistor. *Nature Nano.* **7,** 443-447 (2012).

[12] Jock, R. M., et. al., Probing band-tail states in silicon metal-oxide-semiconductor heterostructures with electron spin resonance. Appl. Phys. Lett. **100**, 023503(2012).

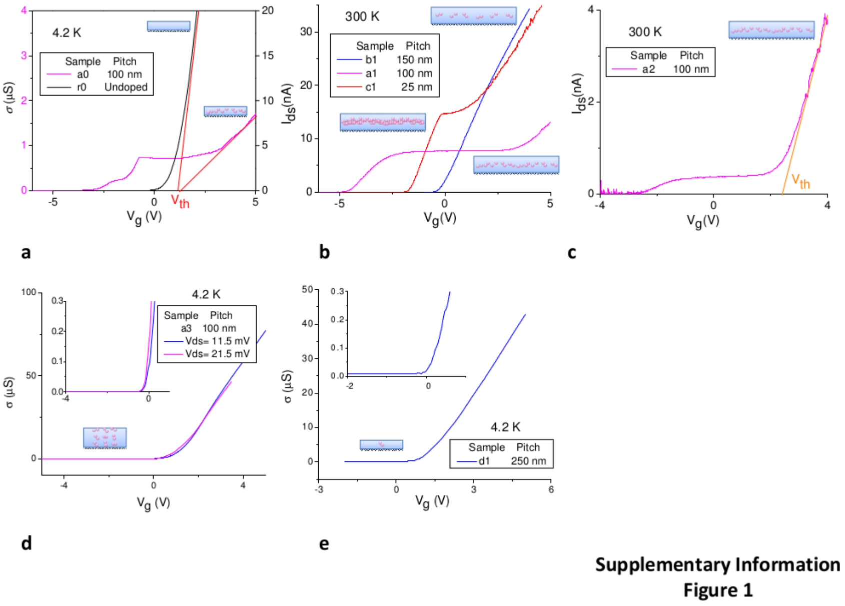

Figure 1: **a** we compare a not-implanted (black, sample r0) and an implanted (violet, sample a0) versions of a device with L = 500 nm and W = 100 nm. The sample a0 is doped at the same 100 nm implantation pitch of main sample a1. The two similar samples have similar threshold voltages V_{th} and are measured at V_{ds} of 11 mV and 15.5 mV respectively, at 4.2 K. Because of the shorter channel (a half of the sample a1 in the main text), 10 P donors (a half) have been implanted in the device by targeting 5 sites along the channel. Consequently, the device falls in the same density regime where impurity bands are formed. The lower conductivity of the implanted device at gate voltages above the threshold voltage with respect to the not-implanted sample are supposed to be determined by the impurity scattering and by the defects caused by the implantation process. The not-implanted sample show neither conductance peaks nor Hubbard bands. **b**: three samples with identical size ($L = 1\mu$m and $W = 200$nm) and different implantation pitch measured at room temperature ($V_{ds} = 2.505$ mV). The sample b1 (pitch of 150 nm, six implant sites for a total of 12 atoms) has an insufficient density to create Hubbard bands and open sub-threshold transport channels. The sample a1 shows the Hubbard band (main text), while the sample c1 (pitch of 25 nm, 40 implant sites) falls in metallic regime, so the 1-dimensional density of states is lost and the flat Hubbard band is replaced by a single metallic impurity band merged with the conduction band [10], reminiscent of the 1-d Hubbard band. The slightly different threshold voltage of the three transistors is due to sample to sample variability before implantation (see [2]). **c** room temperature Hubbard band in the sample a2 nominally identical to a1, measured at $V_{ds} = 2.505$ mV. The different conductance in the flat region is attributed to the sample-to-sample randomness in the coupling between neighboring donors and electron scattering. **d**: sample a3 with same length of a0 ($L = 0.5\mu$m and $W = 250$nm) and same implantation pitch (100 nm) measured at 4.2 K ($V_{ds} = 11.5$ and 22.5 mV). Here, nine implant sites close to the center of the channel for a total of 18 atoms and insufficient distance from the contacts to grant overlap but similar density at the center. The sample a3 shows no-sub threshold voltage current, as expected, even if the same density of samples a0, a1 is achieved, neither the upper Hubbard band nor the conductance peaks. **e**: sample d1 with same size of a0 ($L = 0.5\mu$m and $W = 100$nm) and insufficient implantation pitch (250 nm, namely a single implantation of two P atoms in the center of the device) measured at 4.2 K ($V_{ds} = 6.5mV$). The device d1 is almost undoped and the insufficient distance from the contacts to grant overlap forbids sub-threshold voltage current such as conductance peaks. Like sample r0, the sample d1 is too large to show any conductance peaks determined by quantum dots created by disorder, so only standard I-V curve is observed at 4.2 K.

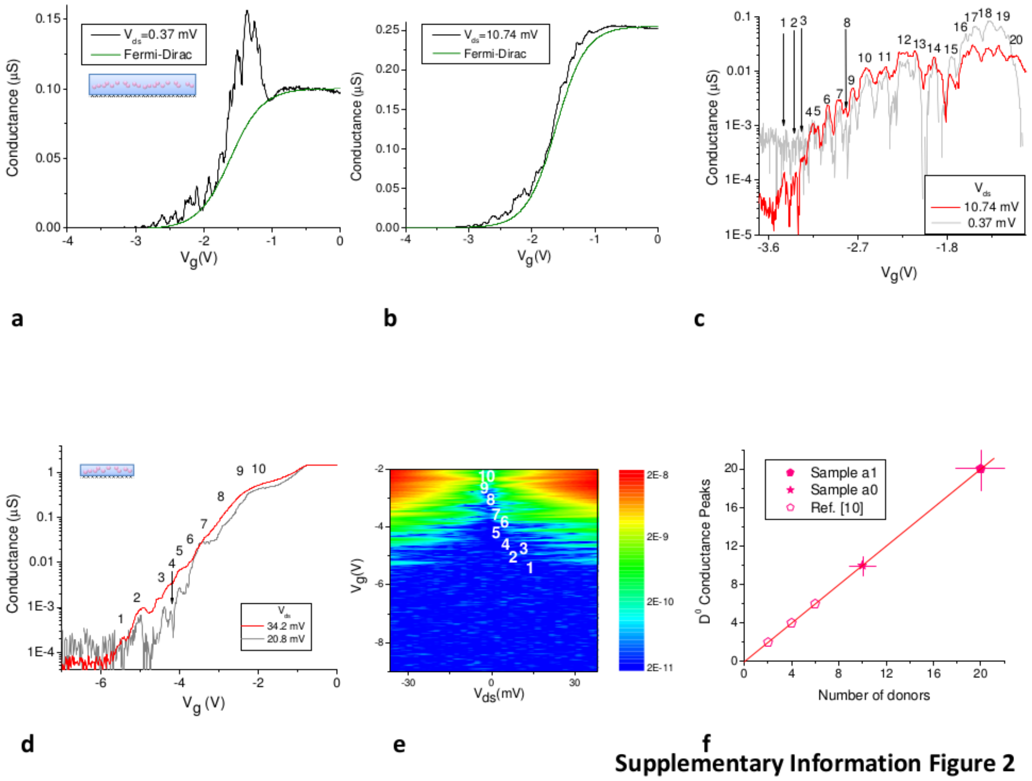

Supplementary Information Figure 2

Figure 2: **a** in order to subtract the base from the transport data to count the conductance peaks of the sample a1, we assumed that the upper Hubbard band is approximated by a Fermi-Dirac equation. The red line shows the Fermi-Dirac fitting curve passing at the minima between conductance peaks. The temperature is 4.2 K and $V_{ds} = 0.37$ mV. **b** by using the same fitting parameters found at the previous point, by only applying a rescaling and an offset, the data at $V_{ds} = 10.74$ mV are matched, by confirming the choice of the fitting function and by better highlighting the peaks at low gate voltage at low electron filling. **c** The 20 conductance peaks of the sample a1 are magnified by subtracting from the conductance the Fermi-Dirac base, at two different drain voltages. The curve at high bias voltage (red) is used to better identify the first peaks, while the curve at low bias (dark grey) voltage is used to count the peaks by excluding possible excited states entering when the bias voltage is high. **d** The conductance peaks of the sample a0 (see SI Figure 1a), partially overlapped with the tail of the upper Hubbard band, at 4.2 K. 10 peaks are observed, corresponding to 10 donors implanted in pairs at the same pitch of sample a1 of main text. **e** Stability diagram of the sample a0 at 4.2 K as alternative view of the 10 peaks reported at the point **d**. **f** The number of conductance peaks at 4.2 K attributed to states formed from D^0 states, as a function of the number of implanted donors. Long arrays (10 and 20 atoms) continue the trend of short arrays of Ref. [11]. The number of conductivity peaks coincides with the number of implanted donors with an uncertainty of 1 and 2 respectively along both the axis. This fact, together with the absence of conductance peaks when the density is insufficient (Figure SI 1b, 1d, 1e) or dopants absent (Figure SI 1a) excludes conduction band localization effects at the interface like those reported in Ref. [12] (which, for an average diameter of 28-37 nm, would produce about 50 localization sites for $W = 100$ nm and $L = 500$ nm of sample a0 and about 200 for $W = 200$ nm and $L = 1000$ nm of sample a1), as as alternative cause of the peaks.
